# Determinants associated with deprivation in multimorbid patients in primary care—A cross-sectional study in Switzerland

**DOI:** 10.1371/journal.pone.0181534

**Published:** 2017-07-24

**Authors:** Silja Leiser, Anouk Déruaz-Luyet, A. Alexandra N’Goran, Jérôme Pasquier, Sven Streit, Stefan Neuner-Jehle, Andreas Zeller, Dagmar M. Haller, Lilli Herzig, Patrick Bodenmann

**Affiliations:** 1 Institute of Family Medicine, University of Lausanne, Lausanne, Switzerland; 2 Institute of Preventive and Social Medicine, University of Lausanne, Lausanne, Switzerland; 3 Institute of Primary Health Care (BIHAM), University of Bern, Bern, Switzerland; 4 Institute of Primary Care, University of Zurich, Zurich, Switzerland; 5 Centre for Primary Health Care, University of Basel, Basel, Switzerland; 6 Primary Care Unit, Faculty of Medicine, University of Geneva, Geneva, Switzerland; 7 Department of Ambulatory Care and Community Medicine, Lausanne University Hospital, Lausanne, Switzerland; TNO, NETHERLANDS

## Abstract

**Background:**

Deprivation usually encompasses material, social, and health components. It has been shown to be associated with greater risks of developing chronic health conditions and of worse outcome in multimorbidity. The DipCare questionnaire, an instrument developed and validated in Switzerland for use in primary care, identifies patients subject to potentially higher levels of deprivation.

**Objectives:**

To identifying determinants of the material, social, and health profiles associated with deprivation in a sample of multimorbid, primary care patients, and thus set priorities in screening for deprivation in this population.

**Design:**

Secondary analysis from a nationwide cross-sectional study in Switzerland.

**Participants:**

A random sample of 886 adult patients suffering from at least three chronic health conditions.

**Main measures:**

The outcomes of interest were the patients’ levels of deprivation as measured using the DipCare questionnaire. Classification And Regression Tree analysis identified the independent variables that separated the examined population into groups with increasing deprivation scores. Finally, a sensitivity analysis (multivariate regression) confirmed the robustness of our results.

**Key results:**

Being aged under 64 years old was associated with higher overall, material, and health deprivation; being aged over 77 years old was associated with higher social deprivation. Other variables associated with deprivation were the level of education, marital status, and the presence of depression or chronic pain.

**Conclusion:**

Specific profiles, such as being younger, were associated with higher levels of overall, material, and health deprivation in multimorbid patients. In contrast, patients over 77 years old reported higher levels of social deprivation. Furthermore, chronic pain and depression added to the score for health deprivation. It is important that GPs consider the possibility of deprivation in these multimorbid patients and are able to identify it, both in order to encourage treatment adherence and limit any forgoing of care for financial reasons.

## Introduction

Multimorbidity is defined as the co-occurrence of two or more chronic medical conditions within one person [1]. With aging populations, the prevalence of multiple chronic medical conditions is increasing worldwide and having a growing impact on healthcare systems [2]. General practice is well-suited to the management of multimorbidity [3]. Several studies have identified deprivation as a predictor of the development and outcome of certain chronic diseases (e.g., heart failure) [4] and of a decreased life expectancy [5].

Deprivation is defined as a manifest material or social disadvantage relative to the local community or society more broadly. Deprivation can be described in material, social, and health terms [6]. It is associated with chronic conditions such as higher cardiovascular risk factors, detrimental lifestyle habits (e.g., excessive alcohol consumption or smoking), and the development of mental health conditions [7,8], among other things. Furthermore, the co-occurrence of two or more chronic medical conditions has been proven to be more prevalent in deprived patients [9]. Salisbury et al. showed that the most deprived patients of general practices in Scotland were almost twice as likely to have multimorbidity than those less deprived [10]. Forgoing healthcare for financial reasons has been demonstrated to lead to a decline in health status [10,11], as well as to cost-related non-adherence to medication [11].

In Switzerland, a country internationally perceived as rich, 7.9% (1 in 12) of the Swiss population lived under the absolute poverty line in 2012 [12]. Despite a mandatory private insurance system for healthcare expenses, out-of-pocket medical spending is still high (4.5% of final household consumption in 2013) [13]. In the population of patients consulting Swiss general practitioners (GPs), 10% forgo healthcare for financial reasons every year (Senn et al., personal communication).

Questionnaires have been developed to facilitate the identification of deprivation [14–16]. Their use aims to alleviate some of the embarrassment that can be associated with direct questions about personal finances while facilitating the gathering of relevant information by the GP. Vaucher et al. developed the DipCare questionnaire (DipCare-q) as a screening instrument for deprivation, specifically adapted to the population of primary care patients in Switzerland [17].

Although multimorbidity and deprivation seem to be associated [9, 10], it is as yet impossible to identify which multimorbid patients at a GP’s practice it would be advisable to evaluate with regard to their level of deprivation.

The present study aimed to identify determinants associated with deprivation in multimorbid patients in primary care. It subsequently aimed to define the patient profiles associated with deprivation in order to set priorities for screening for it in Switzerland.

## Methods

### Study design and participants

We conducted a cross-sectional survey investigating multimorbidity in GPs’ practices. The study protocol has been described elsewhere [18]. In brief, 100 GPs, associated with one of Switzerland’s five university family medicine institutes, randomly enrolled 888 patients between January 12 and September 30, 2015. For the current analyses, missing data were deleted, and the final sample of patients for this study consisted of 886 participants (99.8% of the initial sample).

Data were collected using three questionnaires. The GPs filled out a paper-based questionnaire including personal characteristics such as their gender and age and information about their practice. They completed another paper-based questionnaire describing the number, type, and severity of each enrolled patient’s chronic medical disorders. In parallel, a trained research assistant completed a telephone questionnaire with the patients. Among other variables, this investigated the patient’s level of deprivation using the DipCare-q. The DipCare-q contains 16 questions examining the three dimensions of deprivation: material, social, and health (S1 Table. Dipcare-q). The material deprivation score is the sum of eight items investigating material circumstances such as monthly payments, clothing, or food (Material score 0–8). Social integration is evaluated using five items (Social score 0–5). Finally, the health dimension groups three questions about physical impairments, mental impairments, and problems related to alcohol or substance use and gambling (Health score 0–3). To compute overall deprivation (DipCare score 0–5.4) we used the equation below, as described by Vaucher et al. [17]:

Overall deprivation = 0.810*Material score + 0.455*Social score + 0.711*Health score

Patients were included if they were at least 18 years old and suffered from at least three conditions belonging to a list of 75 chronic medical conditions elaborated by N’Goran et al. [19].

### Ethics

Each participant gave their written informed consent. The Human Research Ethics Committee of the Canton Vaud acted as the lead for the approval of this cross-sectional study (Protocol 315/14).

### Measurements

#### Dependent variables

The overall DipCare score was the primary dependent variable.

The separate sub-scores (material, social, and health scores) for each dimension of the DipCare-q were used as secondary dependent variables.

#### Independent variables

We evaluated GP-related and patient-related variables. We evaluated the association of deprivation with the location of the GP’s practice (‘urban’, ‘suburban’, and ‘rural’). Patient-related variables (as reported by the GPs) were age, gender, and the type, number, and severity of their chronic medical conditions. The severity of chronic conditions was described using the cumulative illness rating scale (CIRS) [20]. The patient’s telephone interview was used to assess patient-related variables such as their marital status (‘single’, ‘married’, ‘divorced’, or ‘widowed’) and level of education (‘primary’, ‘secondary’, or ‘tertiary’).

### Statistical analysis

We used R software, version 3.2.4 (Foundation for Statistical Computing, Vienna, Austria), for the Classification And Regression Tree (CART) analysis and Stata software, version 14.1 (StataCorp LP, College Station, TX, USA), for the remaining analyses.

First, we conducted descriptive analyses, presented as mean ± standard deviation (SD) for normally distributed quantitative variables and as median and 25^th^ and 75^th^ percentiles for non-normally distributed variables. Categorical variables (practice locality, marital status, or level of education) were presented as frequencies.

Second, we conducted a CART analysis to identify which independent variables separated the examined population into groups with increasing deprivation scores and to build a final multivariate model. CART analysis is a statistical method that identifies subgroups of a population which share common characteristics. The visual result of CART analysis resembles a tree with parent-nodes splitting into two child-nodes (subgroups) according to the level of the corresponding independent variable. The further sprouting of branches proceeds, determining for every independent variable, if and how it splits the best according to the splitting criterion [21]. The non-parametric nature of using a CART allows us to analyze non-normally distributed dependent variables. Furthermore, due to its efficient algorithms, an elevated number of independent variables can be analyzed [22]. As the present study analyzed continuous, non-binary data, regression trees were used to identify the splits with maximal R^2^ [23]. This procedure continued through each branch of the tree until a maximal factor of complexity (here 0.2) could no longer be passed. The mean deprivation score was measured at each child node.

Third, we tested the robustness of our results using sensitivity analysis. A linear regression was used for overall deprivation. Ordinal logistic regression models (adjacent categories logit models) were used for the material, social, and health deprivation scores, as the underlying assumptions of the linear regression models were not met for those outcome variables. We assumed that the risk of sliding into a worse category of material, social, or health deprivation, depending on various predictors (independent variables), was the same for all categories of deprivation (hypothesis of proportionality). An iterative selection (step forward) included every predictor-variable that increased the likelihood of sliding into a worse category, at a significant alpha level of 5%. The complete results of the linear and ordinal regressions can be found in the supporting information (S2–S5 Tables).

## Results

### Descriptive analysis

All patients’ and GPs’ characteristics are reported in Table 1. Of the 100 GPs included, 72% were male, with a mean age (± SD) of 52.9 (± 9.3) years old. Thirty-six practiced in urban areas, 44 in suburban areas, and 20 in rural regions of Switzerland.

**Table 1:** Characteristics of patients and GPs.

	Patients *(N = 886)*	GPs *(N = 100)*
Variables	Mean (SD)	Mean (SD)
**Age (years)**	72.9 (12.0)	52.9 (9.3)
**DipCare score**	1.2 (0.9)	-
	**N (%)**	**N (%)**
**Gender**		
Male	427 (48.2)	72 (72)
Female	459 (51.8)	28 (28)
**Locality of practice**		
Urban	-	36 (36
Suburban	-	44 (44)
Rural	-	20 (20)
**Marital status**		
Single N (%)	85 (9.6)	-
Married N (%)	436 (49.2)	-
Divorced N (%)	149 (16.8)	-
Widow N (%)	216 (24.4)	-
**Level of education**		
Primary (compulsory school)	194 (21.9)	-
Secondary (practical, high school)	337 (38.0)	-
Tertiary (university, college)	355 (40.1)	-
	**Median (Q1, Q3)**	-
**Number of chronic conditions**	5 (4, 6)	**-**
**Total CIRS score**	10 (7, 13)	**-**

SD = standard deviation

Q1, Q3 = 25^th^ percentile and 75^th^ percentile

Of the 886 patients included, 48.2% were male, with a mean age of 72.9 (± 12.0) years old. The majority of the participants (49.2%) were married, and 40.1% had a college or university level of education. The median number of chronic conditions was 5 (Q1: 4, Q3: 6), and the median total CIRS score was 10 (Q1: 7, Q3: 13).

Detailed results from the DipCare-q are reported in Table 2. The mean overall DipCare score is a computed score ranging from 0 to 5.4. The population examined had a mean DipCare score of 1.2 ± 0.9.

**Table 2:** Results DipCare-q.

Dimensions	Items (Item 1–16)	Frequency (%) *(N = 886)*
**Material**	I1: Difficulties paying bills	104 (11.7)
	I2: Need to borrow money for daily expenses	52 (5.9)
	I3: Forgoing healthcare	30 (3.4)
	I4: Scared of losing housing	34 (3.8)
	I5: Cannot afford clothes	81 (9.1)
	I6: Cannot afford furniture	72 (8.1)
	I10: Not enough to eat at home	11 (1.2)
	I13: Difficulties reimbursing loans	53 (6.0)
**Social**	I7: No holidays	453 (51.1)
	I8: No evenings spent with family or friends	115 (13.0)
	I9: No cultural activities	478 (54.0)
	I11: No access to the internet	389 (43.9)
	I12: No one to turn to for material support	279 (31.5)
**Health**	I14: Physical handicap	274 (30.9)
	I15: Psychological handicap	118 (13.3)
	I16: Addiction	27 (3.0)

Frequency of positive responses for each DipCare-q item among 886 multimorbid patients attending GP practices.

The proportion of patients reporting some level of material deprivation (items 1–6, 10, and 13) varied greatly, depending on the question, from 1% (minimum) of patients reporting difficulties paying bills to 12% (maximum) not being able to afford clothes or food (Table 2).

With regard to social deprivation (items 7–9, 11, and 12), scores ranged from 30% (minimum) to 50% (maximum) of subjects reporting having no access to internet, not going out or on holidays, not spending evenings with close friends, or having nobody outside the family to turn to in situations of need.

Regarding health deprivation (items 14–16), 30% of patients reported a physical disability, 13% reported a psychiatric disorder, and 3% reported abuse of alcohol or illicit substances or gambling issues.

### CART analysis and linear and ordinal regression

**Fig 1:**
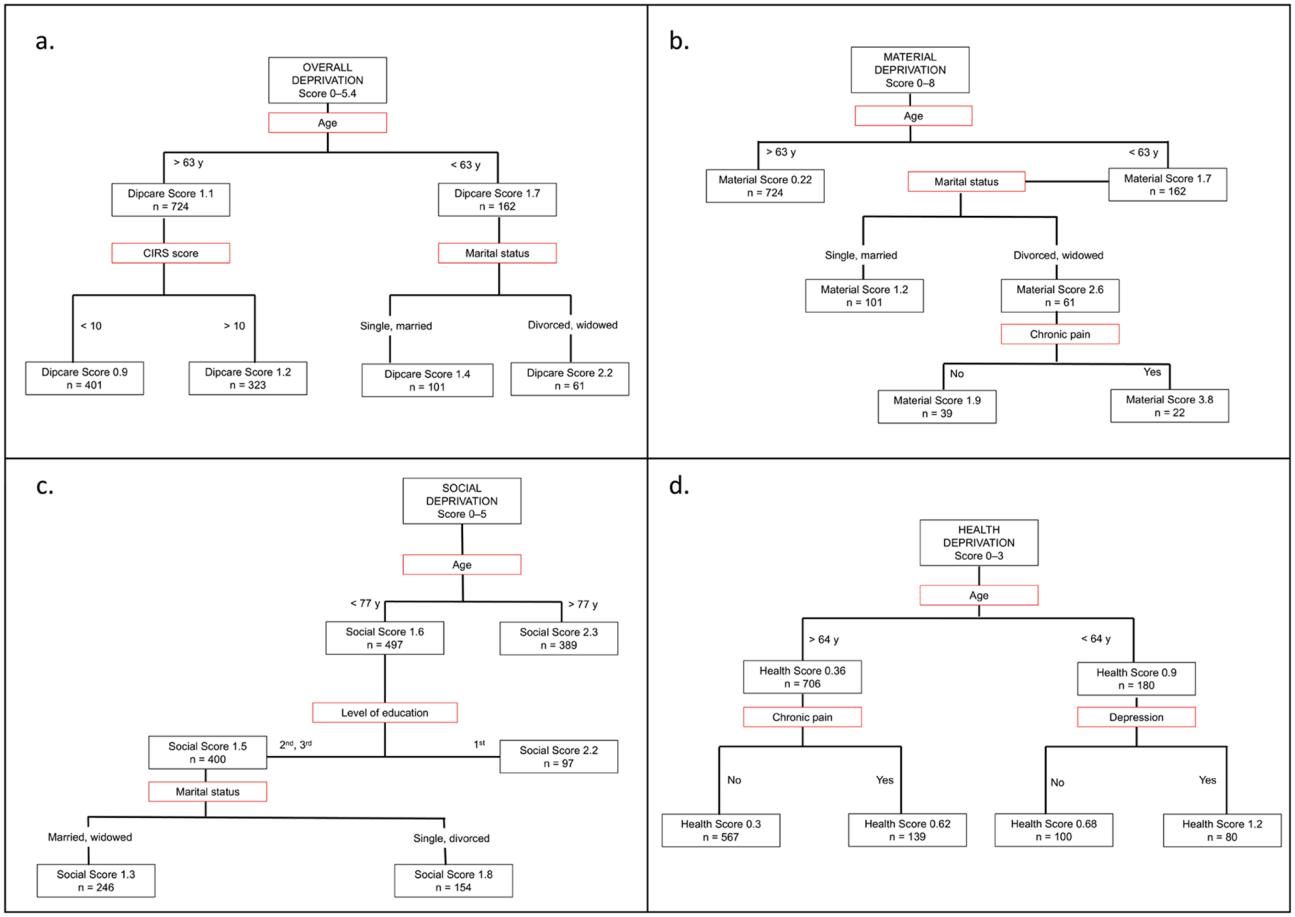
CART analysis. Classification And Regression Tree analysis of variables separating the examined population into subgroups with increasing overall (a.), material (b.), social (c.) and health (d.) deprivation scores.

The results of the CART analysis for deprivation are shown in Fig 1.

#### Overall deprivation

The first variable in the CART analysis shown to segregate the population into different degrees of deprivation was the patient’s age. The greatest overall deprivation score (mean DipCare score: 2.2) was associated with the subgroup of patients aged less than 63 years old and divorced or widowed. The least deprived subgroup (mean DipCare score: 0.9) identified in CART analysis was of patients over 63 years old and with a CIRS score under 10.

#### Material deprivation

Material deprivation score was highest (mean: 3.8) in patients under 63 years old, divorced or widowed, and in chronic pain. Being over 63 years old was associated with lower material deprivation scores (mean: 0.22).

#### Social deprivation

Patients over 77 years old had higher social deprivation scores (mean: 2.3). The age group under 77 years old, with a secondary or tertiary level of education, and either married or widowed, showed the lowest social deprivation scores (mean: 1.3). The next lowest subgroup comprised patients under 77 years old, with a secondary or tertiary level of education, and either single or divorced (mean social score of 1.8). Finally, subjects with a level of education limited to compulsory schooling had a mean social score at 2.2.

The nine patients under 77 years old suffering from memory loss had the highest social deprivation score (mean: 3.3), but were not considered because of their small number.

#### Health deprivation

CART analysis revealed that health deprivation was worst for patients under 64 years old and suffering from depression (mean health score: 1.2). The subgroup over 64 years old and free of chronic pain had the lowest health deprivation score (mean: 0.3).

#### Sensitivity analysis

The complete results of the multivariate regression analysis can be found in the supporting information. (S2–S5 Tables).

Sensitivity analysis confirmed all the CART results with significant associations, especially associations between all the categories of deprivation and age, between the level of education and social deprivation, and between chronic pain or depression and health deprivation. Only the associations between overall, material, and social deprivation and marital status were less robust, as the association with deprivation was not significant for any of the four categories of marital status.

## Discussion

This study aimed to identify variables associated with deprivation in a sample of multimorbid patients consulting at GPs’ practices. Age was identified as a key parameter associated with deprivation. Both overall and material deprivation scores, and health deprivation scores increased for multimorbid patients under the ages of 63 and 64 years old, respectively. Social deprivation increased in subjects over 77 years old. Other variables associated with deprivation were the level of education, marital status, and the presence of depression or chronic pain.

Patients under 63 years old reported higher material deprivation. This might be related to specifics of the Swiss social security system favoring old age, as shown by the Swiss Federal Office of Health [24]. Indeed, in Switzerland, retired persons (women over 64 and men over 65 years old) benefit from a monthly pension income from their retirement fund and, very often, from reduced healthcare insurance premiums.

Being under 64 years old was also related to higher levels of health deprivation. This may seem contradictory as with increasing age, the number of health problems increases [25]. However, in the present study, health deprivation was not narrowly defined as a physical handicap; it also included the potential presence of an addiction or a mental disorder. These health conditions have been shown to be more frequent in younger adults and are proportionally more frequently associated with physical multimorbidity [26] and, therefore, health deprivation.

Indeed, co-occurring chronic conditions are not only present in old age, as shown by the studies of Van den Akker [25], Barnett [26], and Taylor [27]. In total, 23% (204) of study participants were under 65 years old, underlining the presence of multimorbidity in younger populations. Interestingly, Barnett et al. identified overall deprivation as a potential factor associated with multimorbidity in patients in this younger age group. In that study, the age of multimorbid patients was also lower if the multimorbidity consisted of physical—mental comorbidity [26].

Our study showed higher levels of social deprivation in patients aged over 77 years old. Van den Akker provided a probable explanation for this, associating the rising prevalence of chronic medical conditions and, therefore, decreased mobility and independence, with increasing age [25]. Marengoni et al. confirmed the associations of multimorbidity with restricted mobility and elevated social isolation [28]. They also showed the association of dementia and/or sensory impairment (e.g., blindness or deafness) with increasing social isolation [28].

Marmot et al. established education as a social determinant of wealth and health [29]. Ross et al. described the association between a higher level of education and professional outcomes, financial income, and social support [30]. The present research is in line with these studies, showing lower scores for social deprivation in multimorbid patients with levels of education beyond primary school.

Furthermore, our study identified chronic pain and depression as being associated with deprivation, especially health deprivation (i.e., physical or mental impairment and addiction).

Chronic pain in patients under 63 years old was associated with higher levels of material deprivation. Blyth et al. demonstrated that the incapacity to work, as a consequence of chronic pain, resulted in lower incomes [31].

On the other hand, the present study associated chronic pain in patients over 64 years old with higher levels of health deprivation. This association may be in line with the explanation by Breivik et al. that chronic pain causes social limitations and financial demands (e.g., costs of medication and home care) and thus leads to physical and/or mental impairment [32].

Depression was associated with increased health deprivation in patients under 64 years old. Mental illness has indeed been shown to be frequent in multimorbid patients; even more so for the young and deprived [26]. This fact was evaluated in a large meta-analysis by Egan et al. [33], yet no causal link could be shown.

The identification of deprivation has practical implications for GPs’ everyday consulting, as it is related both to a higher prevalence of the co-occurrence of chronic conditions [9] and to worse outcomes in cases of multimorbidity [4,5]. Hardee et al. showed that talking about treatment costs and the patient’s socioeconomic situation reinforces the empathetic relationship between the GP and the patient and stimulates conscious decisions and better adherence to treatment [34]. However, even if patients discuss personal financial matters, GPs either underestimate poverty or try to avoid embarrassing their patients by evading cost-related questions, as Bloch and Chatelard et al. have described [35,36]. Using the determinants described in the present study, GPs could more easily identify which patients were prone to material, social, and health deprivation. For example, item 1 (“During the last 12 months, have you had trouble paying your household bills?”), which has been shown to be sensitive in evaluating the risks of subjects forgoing healthcare [37], could be used as a screening question to examine the risk of patients forgoing healthcare for financial reasons, associated with an even greater occurrence of chronic disorders and a worse general health status [38].

### Strengths and limitations

First, due to its cross-sectional design, the present study’s conclusions were limited to associations without predictive value. Although a causal link cannot be proven, it still seems important to explore any association between multimorbidity and deprivation which might help GPs identify patients who may be suffering from deprivation. Further studies should concentrate on discovering any causal links between deprivation and multimorbidity, thus providing a better chance of early prevention.

Second, there is no clearly defined cut-off value for the DipCare score. Therefore, a quantitative prevalence of deprivation could not be defined for this study.

Third, the present study is a secondary analysis of variables chosen for a study of multimorbidity. We therefore had no influence on the content of those variables. The inclusion of a large panel of patients, spread across Switzerland, nevertheless increased the statistical significance of our results.

Finally, we cannot exclude a potential selection bias in our study’s population as we find a high percentage of married multimorbid patients with high levels of education and a high mean age. Unfortunately, comparison with our cross-sectional multicentric survey of deprivation in the French part of Switzerland [36] or with the federal office of Swiss statistics was not possible as all necessary variables are not available. However, we think that the inclusion of patients by 100 GPs in the whole area of Switzerland is representative of the characteristics of a multimorbid population in this setting. Further studies may better take in account this result.

## Conclusion

In a sample of multimorbid patients in a primary-care setting, we found that age played a differential role in identifying deprivation: multimorbid patients under 64 years old reported higher overall, material, and health deprivation. In contrast, patients over 77 years old reported higher levels of social deprivation. Furthermore, chronic pain and depression added to the score for health deprivation. These results imply that GPs should consider the possibility of deprivation when treating multimorbid patients. For the future, we recommend the development of more sophisticated tools for the identification of patients prone to deprivation as well as interventions that help increase treatment adherence and limit any forgoing of healthcare for financial reasons.

## Supporting information

S1 Table DipCare-q. (PDF)

S2 Table Sensitivity analysis of overall deprivation. (PDF)

S3 Table Sensitivity analysis of material deprivation. (PDF)

S4 Table Sensitivity analysis of social deprivation. (PDF)

S5 Table Sensitivity analysis of health deprivation. (PDF)
