# Supplementary material for: Determinants associated with deprivation in multimorbid patients in primary care—A cross-sectional study in Switzerland
Source: PLoS One. 2017 Jul 24;12(7):e0181534. doi: 10.1371/journal.pone.0181534 (PMC5524289; doi:10.1371/journal.pone.0181534)
Supplement: S1 Table — (PDF) [file pone.0181534.s001.pdf]

S1 Table. DipCare-q

1) During the last 12 months, have you had trouble paying your household bills (taxes, insurance, telephone, electricity, credit cards, etc.)?
2) During the last 12 months, have you had to ask your immediate family for money to cover your basic day-to-day needs?
3) During the last 12 months, has a member of your household not sought treatment (dentist, doctor, buying medication) because you did not have enough money?
4) During the last 12 months, have you feared being evicted from or losing your home?
5) During the last 12 months, have you not bought clothes even though you or a member of your household needed them?
6) During the last 12 months, have you not bought furniture or household goods even though you or a member of your household needed them?
7) During the last 12 months, have you gone on holiday?
8) During the last 3 months, have you spent an evening in the company of close family members or friends?
9) During the last 3 months, have you been to the cinema, the theatre, a concert, or a sports event?
10) During the last month, has there been an occasion when your household did not have enough to eat?
11) During the last month, have you been able to access the internet (at home, at work, at a library, at an internet café, etc.)?
12) If you are in difficulty, is there someone outside your household to whom you can turn for material help (money, food, accommodation)?
13) Are you currently finding it very difficult to pay back money (to the bank, family, a friend, etc.)?
14) Do you currently suffer from a physical disability that has a major impact on your day-to-day life?
15) Do you currently suffer from mental health issues or problems that have a major impact on your day-to-day life?
16) Do you currently have problems linked to alcohol consumption, drug-taking, gambling, etc.?
