# Supplementary material for: Determinants associated with deprivation in multimorbid patients in primary care—A cross-sectional study in Switzerland
Source: PLoS One. 2017 Jul 24;12(7):e0181534. doi: 10.1371/journal.pone.0181534 (PMC5524289; doi:10.1371/journal.pone.0181534)
Supplement: S2 Table — (PDF) [file pone.0181534.s002.pdf]

S2 Table. Sensitivity analysis of overall deprivation

Overall deprivation	Coef. (Q1, Q3)	p-value
**Age**	-0.11 (-0.16, -0.05)	0.00*
**Marital status**		
Married	-0.20 (-0.39, 0.00)	0.05*
Divorced	0.27 (0.05, 0.50)	0.02*
Widowed	0.11 (-0.12, 0.33)	0.35
**Level of education**		
Secondary	-0.26 (-0.41, -0.11)	0.00*
Tertiary	-0.33 (-0.47, -0.18)	0.00*
**Locality of practice**		
Suburban	-0.13 (-0.25, 0.00)	0.05*
Rural	-0.16 (-0.32, -0.01)	0.04*
**Total CIRS score**	0.03 (0.01, 0.04)	0.00*
**Number of conditions**	0.06 (0.03, 0.09)	0.00*
**Pain A01**	0.18 (0.04, 0.32)	0.01*
**IBS D93**	-0.23 (-0.43, -0.04)	0.02*
**Blindness F94**	0.56 (0.07, 1.06)	0.03*
**Cardiovasc. RF K22**	-0.27 (-0.39, -0.15)	0.00*
**Arthritis L88**	-0.27 (-0.48, -0.05)	0.02*
**Drug abuse P19**	0.76 (0.21, 1.32)	0.01*
**Affect. psychosis P73**	0.70 (0.20, 1.20)	0.01*
**Asthma R96**	-0.34 (-0.53, -0.14)	0.00*
**Obesity T71**	-0.81 (-1.62, 0.00)	0.05*

* = significant; □variables in CART; Q1, Q3 = 25th percentile and 75th percentile
