# Supplementary material for: Determinants associated with deprivation in multimorbid patients in primary care—A cross-sectional study in Switzerland
Source: PLoS One. 2017 Jul 24;12(7):e0181534. doi: 10.1371/journal.pone.0181534 (PMC5524289; doi:10.1371/journal.pone.0181534)
Supplement: S3 Table — (PDF) [file pone.0181534.s003.pdf]

S3 Table. Sensitivity analysis of material deprivation

**Material deprivation**	**Coef. (Q1, Q3)**	**p-value**
**Age**	0.70 (0.65, 0.76)	0.00*
**Marital status**		
Married	1.05 (0.81, 1.35)	0.73
Divorced	1.74 (1.35, 2.24)	0.00*
Widowed	1.53 (1.12, 2.09)	0.01*
**Number of conditions**	1.05 (1.02, 1.08)	0.00*
**Pain A01**	1.20 (1.04, 1.40)	0.01*
**IHD**	1.25 (1.05, 1.49)	0.01*
**Affect. psychosis P73**	1.63 (1.13, 2.35)	0.01*
**Depressive disord. P76**	1.20 (1.04, 1.40)	0.01*
**Asthma R96**	0.80 (0.63, 1.01)	0.07

* = significant; □variables in CART; Q1, Q3 = 25th percentile and 75th percentile
