# Supplementary material for: Determinants associated with deprivation in multimorbid patients in primary care—A cross-sectional study in Switzerland
Source: PLoS One. 2017 Jul 24;12(7):e0181534. doi: 10.1371/journal.pone.0181534 (PMC5524289; doi:10.1371/journal.pone.0181534)
Supplement: S4 Table — (PDF) [file pone.0181534.s004.pdf]

S4 Table. Sensitivity analysis of social deprivation

**Social deprivation**	**Coef. (Q1, Q3)**	**p-value**
**Age**	1.14 (1.08, 1.20)	0.00*
**Marital status**		
Married	0.76 (0.63, 0.93)	0.01*
Divorced	0.98 (0.79, 1.22)	0.86
Widowed	0.93 (0.75, 1.16)	0.52
**Level of education**		
Secondary	0.73 (0.63, 0.84)	0.00*
Tertiary	0.67 (0.58, 0.77)	0.00*
**Total CIRS score**	1.03 (1.01, 1.04)	0.00*
**Number of conditions**	1.03 (1.00, 1.06)	0.06
**Blindness F94**	1.72 (1.07, 2.77)	0.02*
**Hearing compl. H02**	0.83 (0.69, 0.98)	0.03*
**Cardiovasc. RF K22**	0.79 (0.70, 0.89)	0.00*
**Arthr. hip L89**	1.21 (1.04, 1.40)	0.01*
**Arthr. knee L90**	0.84 (0.74, 0.96)	0.01*
**Drug abuse P19**	1.82 (1.07, 3.10)	0.03*
**Depressive disord. P76**	1.15 (1.01, 1.31)	0.04*
**Psychosis P98**	2.55 (1.12, 5.81)	0.03*
**Asthma R96**	0.74 (0.60, 0.90)	0.00*
**Skin ulcer S97**	1.52 (1.12, 2.06)	0.01*

* = significant; □variables in CART; Q1, Q3 = 25th percentile and 75th percentile
