# Supplementary material for: Determinants associated with deprivation in multimorbid patients in primary care—A cross-sectional study in Switzerland
Source: PLoS One. 2017 Jul 24;12(7):e0181534. doi: 10.1371/journal.pone.0181534 (PMC5524289; doi:10.1371/journal.pone.0181534)
Supplement: S5 Table — (PDF) [file pone.0181534.s005.pdf]

S5 Table. Sensitivity analysis of health deprivation

Health deprivation	Coef.	p-value
**Age**	0.73 (0.65, 0.82)	0.00*
**Marital status**		
Married	0.78 (0.53, 1.16)	0.23
Divorced	1.30 (0.85, 1.98)	0.23
Widowed	0.86 (0.54, 1.37)	0.52
**Locality of practice**		
Suburban	0.94 (0.73, 1.22)	0.65
Rural	0.66 (0.47, 0.94)	0.02*
**Total CIRS score**	1.06 (1.03, 1.09)	0.00*
**Number of conditions**	1.08 (1.01, 1.14)	0.02*
**Pain A01**	1.75 (1.34, 2.30)	0.00*
**Trauma A82**	2.37 (1.34, 4.21)	0.00*
**High blood press. BP**	0.67 (0.51, 0.88)	0.00*
**IBS D93**	0.64 (0.42, 0.97)	0.04*
**Retinopathy F83**	0.28 (0.10, 0.82)	0.02*
**Arthr. knee L90**	1.27 (0.97, 1.67)	0.08
**Osteoporosis L95**	1.35 (1.00, 1.82)	0.05*
**Alcohol abuse P15**	1.76 (1.12, 2.76)	0.01*
**Affect. psychosis P73**	4.15 (1.80, 9.55)	0.00*
**Depressive disord. P76**	1.79 (1.38, 2.31)	0.00*
**Chronic bronchitis R79**	0.45 (0.23, 0.87)	0.02*
**Asthma R96**	0.68 (0.45, 1.01)	0.06

* = significant; □variables in CART; Q1, Q3 = 25th percentile and 75th percentile
